# Social engagement before and after dementia diagnosis in the English Longitudinal Study of Ageing

**DOI:** 10.1371/journal.pone.0220195

**Published:** 2019-08-01

**Authors:** Ruth A. Hackett, Andrew Steptoe, Dorina Cadar, Daisy Fancourt

**Affiliations:** Department of Behavioural Science and Health, University College London, London, United Kingdom; Cardiff University, UNITED KINGDOM

## Abstract

**Background:**

Social engagement protects against dementia onset. Less is known about patterns of social engagement around the time of dementia diagnosis. We investigated face-to-face and telephone contact at three times (pre-diagnosis, at report of diagnosis, 2 years post-diagnosis) in individuals who developed dementia and a comparison group.

**Methods:**

Social engagement was assessed at waves 2–7 of the English Longitudinal Study of Ageing in 4171 individuals aged 50 and older. Dementia was ascertained by either self-reported physician diagnosis or through an informant evaluation of a participant’s functional and cognitive performance compared with a few years earlier. Generalized estimating equations were used to examine differences by group, time, and group-by-time interactions.

**Results:**

The dementia group reported less face-to-face (*p* < 0.001) and telephone contact (*p* < 0.001) than the dementia-free group pre-diagnosis. The dementia group experienced greater reductions in social engagement leading up to dementia diagnosis and in the 2 years following diagnosis (*p’s* < 0.001).

**Conclusion:**

Given that social engagement reduces dementia risk and supports the lived experience of people with dementia, it is important to find ways of promoting social interaction in older adults.

## 1. Introduction

Social connections are strongly linked with dementia. Loneliness and isolation are identified predictors of cognitive decline and dementia onset, while social engagement appears to protect against dementia onset [1]. Specifically, these social factors appear to act protectively through increasing cognitive reserve and mental resilience and reducing psychological stress thereby reducing important vascular risk factors for dementia [1]. Neurobiology studies have suggested that loneliness and isolation are also associated with brain activation at both cellular and molecular levels [2]including increased hippocampal neurogenesis, reduced levels of brain-derived neurotrophic factors (BDNF) and increased oxidative stress [3,4]. However, what remains less well understood is the profile of social engagement amongst individuals diagnosed with dementia. Do people with and without dementia have different levels of social interaction with friends and family? And how do profiles of social interaction vary in the years preceding dementia diagnosis and the years immediately after?

It is well known that dementia can affect *cognitive processes* involved in social behaviors. For example, impairments in the level of social interaction and emotion recognition have been traced to mild cognitive impairment [5]. And once people have developed dementia, studies have found impairments in social cognition, language and memory [6–8]. It is also well known that there are changes in *individual behaviors* related to social engagement surrounding dementia onset, including changes in personal attributes (such as apathy, disinhibition and agitation) [9], and the development of impairments in instrumental activities of daily living connected to social engagement such as using a telephone and using transport independently [10]. Notably, these changes have been found as long as 10 years prior to the onset of dementia [10].

However, far fewer studies have focused on *social engagement* itself. One study interviewing carers of people with dementia found that social avoidance was reported in around one third of dementia patients, while a loss of interest was reported in around half of Alzheimer’s disease (AD) and cerebrovascular dementia patients [8]. Further, a previous study of social contact amongst individuals living in a nursing home demonstrated that contact decreased by approximately half following admission [11]. But it remains unclear how social engagement is affected in the years immediately surrounding dementia diagnosis for individuals living in the community. Therefore, this study aimed to characterize social engagement amongst those with dementia in the years before and after diagnosis in comparison with healthy older individuals from an English nationally representative sample. Specifically, we assessed face-to-face and telephone contact at three time points: 2 years before dementia ascertainment, when dementia was first reported and 2 years following dementia ascertainment.

## 2. Materials and methods

### 2.1 Study population

The study data come from the English Longitudinal Study of Ageing (ELSA); a representative study of community-dwelling English adults aged 50 and over [12]. Data collection began in 2002–2003 (wave 1) with follow-ups biennially. A total of 9264 participants took part in the study at wave 2 (2004–5) which serves as the baseline for this study. The latest data available at the time of these analyses was wave 7 (2014–2015). Ethical approval was obtained from the National Research Ethics Committee. All study methods were performed in accordance with the Helsinki Declaration and good clinical and scientific practice. Data was collected through face-to-face interview with the participant or via a nominated informant. Those who did the face-to-face interview gave informed consent. For those who were not able to consent themselves a committee (established through the National Research Ethics Service) approved their participation if they had previously nominated a proxy informant to assist in the completion of measures.

### 2.2 Participants

**Fig 1:**
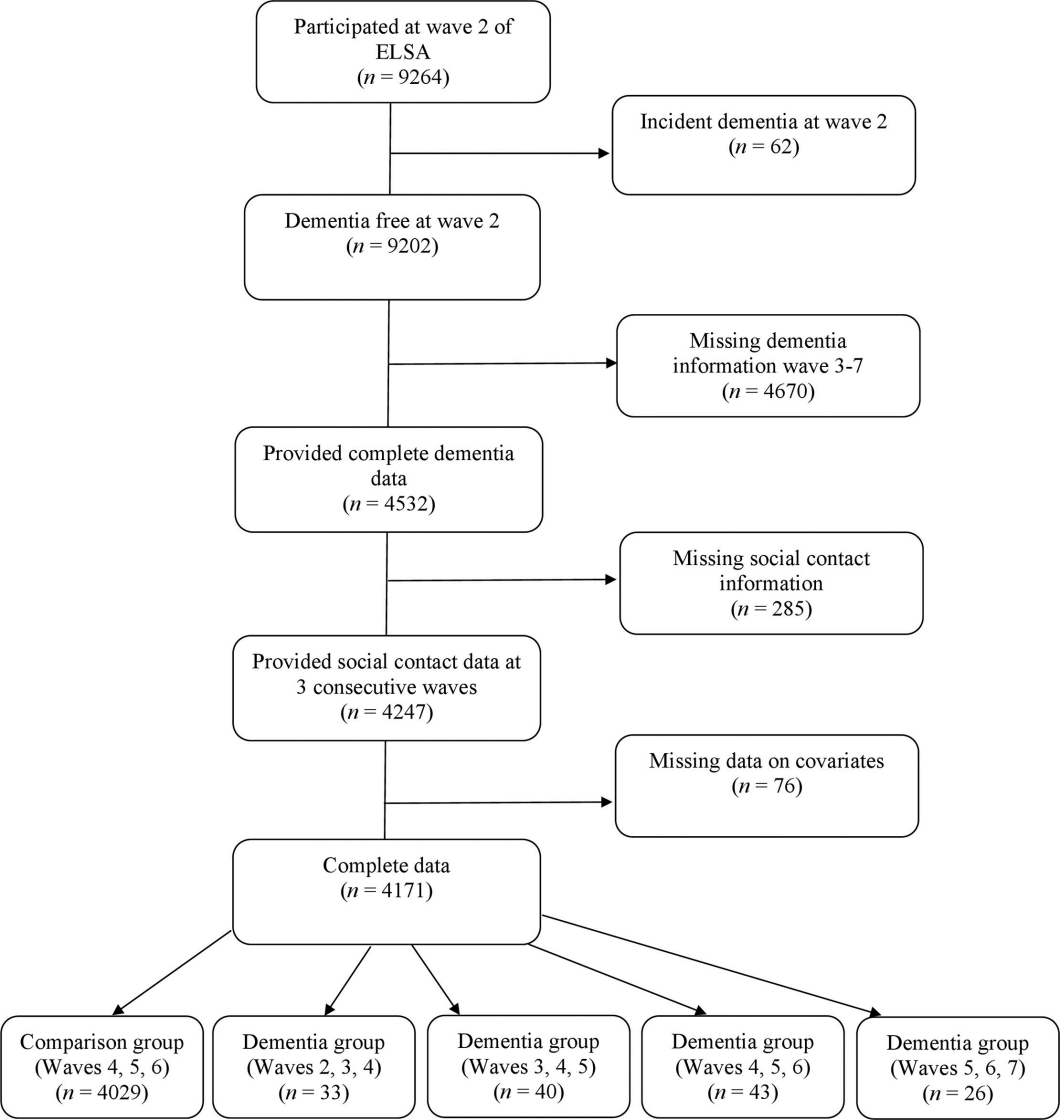
Flow diagram of participants included and excluded from the analyses.

The dementia group consisted of participants who were free of dementia at wave 2 (2004–2005) and reported a new occurrence of dementia at any subsequent wave: wave 3 (2006–2007), wave 4 (2008–2009), 5 (2010–2011) or 6 (2012–2013). For these analyses, we used three different time points: the wave preceding a new report of dementia as “pre-diagnosis” (T0), the wave when dementia was ascertained as “peri-diagnosis” (T1) and the wave following dementia ascertainment as “post-diagnosis” (T2). Individuals with dementia at wave 7 (2014–2015) were excluded due to the absence of post-diagnosis data. The non-dementia (comparison) group consisted of participants who did not report dementia in any wave. Data from waves 4, 5 and 6 were used to identify an equivalent 3-wave period for the non-dementia group. For both groups, only individuals with information on social engagement at three consecutive waves for at least one type of engagement and full information on covariates were included in the study. This resulted in a final sample size of 4171 participants, of whom 142 developed dementia. A flowchart of those included and excluded from the study can be found in Fig 1. The final sample differed significantly from those who were dementia free at wave 2 but were not included in the current analysis (n = 5031). Our sample was significantly younger (63.48 vs 68.75 years), more likely to be female (57.1% vs 54.7%) and were wealthier and better educated than those not included in the study (*p*’s < 0.023).

### 2.3 Dementia definition

Two sources of information were used to define dementia, as in earlier work [13]. The first criterion was self-reported physician-diagnosed dementia. The second indicator was used was the Informant Questionnaire on Cognitive Decline in the Elderly (IQCODE) [14]. This is a standardized scale used in clinical settings to ascertain a participant’s functional and cognitive performance compared with a few years earlier provided by a carer or informant. We used the 16-item adapted short-form version with a modified period covering the last two years instead of the previous ten years as in the original questionnaire. Those with an average score ≥3.38 were defined as dementia cases, as presented in previous work [15]. If a person had either a self-reported physician diagnosis of dementia or a score ≥3.38 on the IQCODE they were classified as a dementia case for this study.

### 2.4 Social engagement

Two measures of social engagement between waves 2 (2004–2005) and 7 (2014–2015) were included in the present study. The first measure was whether the participant reported meeting friends or relatives over the past month (yes/no). The second measure was whether the participant reported speaking with friends or relatives over the telephone in the past month (yes/no). The assessment of social engagement was via a self-completion questionnaire with assistance from an interviewer. This process was the same for participants in the dementia group and the comparison group.

### 2.5 Covariates

Both dementia prevalence and engagement in social activities are age, sex and socially patterned [16,17]. Therefore age, sex, education and household non-pension wealth at “pre-diagnosis” (T0) were included as covariates in all analyses. Age was entered as a continuous variable and sex was a binary variable (male/female). We divided education into four categories: no formal qualifications, O-level (junior high certificate), A-level (high school certificate) and higher education (university degree). Non-pension wealth is a robust indicator of socio-economic status in older age groups[18] and is presented in quintiles (1 = low, 5 = high) in the current study. For sensitivity analyses, depression and limiting standing illness were entered as additional covariates. Depression was measured using the Center for Epidemiological Studies Depression Scale (CES-D)[19], with the total score ranging from 0–8 with higher scores meaning greater depressive symptoms. The limiting longstanding illness measure was a binary variable (yes/no) based on a self-report of any long-standing condition that participants classed as limiting their activities[20]. The procedure for collecting information on covariates was the same for participants with and without dementia.

### 2.6 Statistical analysis

Demographic characteristics of dementia and non-dementia groups at pre-diagnosis (T0) were assessed using t-tests for continuous variables and chi-squared tests for categorical variables. For the longitudinal analyses, generalized estimating equation (GEE) models were used to examine the main effects of group (differences in the prevalence of each type of social engagement between dementia vs control groups independent of time), the main effects of time (changes in social engagement over time independent of group), and the group-by-time interactions (differences in social engagement over time between groups). We considered the demographic differences in age, sex, education and wealth between those in dementia and non-dementia groups, and therefore included them as covariates. We also took account of non-response related to dementia diagnosis by using inverse probability weighting based on propensity scores derived from age, sex, education and wealth variables [21]. The GEE results presented in the current study are based on an unstructured correlation matrix. Analyses using autoregressive, exchangeable or m-dependent matrices did not alter the pattern of results and are therefore not presented. All analyses were conducted using SPSS version 24 and Stata version 14.

### 2.7. Sensitivity analyses

To test the robustness of our findings, we conducted a number of different sensitivity analyses. Dementia onset before the age of 65 is uncommon [16]. Therefore, we conducted a sensitivity analysis to ascertain whether the pattern of results remained the same when excluding younger participants (aged ≤ 63 years at T0) from the analysis. Secondly, considering that depression and illnesses other than dementia may impact social engagement [22,23], we repeated our analyses including depression scores and limiting longstanding illness at baseline as additional covariates. Finally, we tested whether the findings remained the same using a continuous measure for the frequency of engagement. Specifically, we assessed meeting and telephoning friends or relatives on a scale from 1 = no contact, 2 = weekly 3 = every few months and 4 = on a weekly basis. For this analysis, we used repeated measures analysis of variance with time (pre-diagnosis contact, peri-diagnosis contact and post-diagnosis contact) as the within-subjects variable and dementia case as the between-subjects variable controlling for age, sex, wealth and education.

## 3. Results

### 3.1 Descriptive characteristics

The participant characteristics of the healthy participants and those with dementia at the pre-diagnosis stage (T0) are presented in Table 1. Those who developed dementia were significantly older on average (*p <* 0.001), were less educated (*p <* 0.001) and had lower non-pension wealth (*p <* 0.001) than those in the non-dementia group. There was no significant group difference by gender (*p* = 0.74). The proportion of participants engaging socially with others on a monthly basis over the study period are presented in Table 2. For face-to-face contact and telephone contact, the dementia group had lower levels of activity at each time point than the non-dementia group.

**Table 1:** Participant characteristics at the pre-diagnosis stage.

	Dementia group (n = 142)	Comparison group (n = 4029)	p value
Age (years)	78.32 (0.50)	64.89 (0.13)	**< 0.001**
Sex (% men)	59 (41.5%)	1730 (42.9%)	= 0.74
Education (%)			**< 0.001**
No formal education	79 (55.6%)	1129 (28%)	
O-level (Junior high)	18 (12.7%)	778 (19.3%)	
A-level (High school)	32 (22.5%)	1367 (33.9%)	
University degree	13 (9.2%)	755 (18.7%)	
Wealth quintiles (%)			**< 0.001**
1 (lowest)	41 (28.9%)	587 (14.6%)	
2	36 (25.4%)	677 (16.8%)	
3	32 (22.5%)	845 (21%)	
4	16 (11.3%)	900 (22.3%)	
5 (highest)	17 (12%)	1020 (25.3%)	

Data are presented as means (standard deviations) and N (%)

Note: The pre-diagnosis wave (T0) for the comparison group was wave 4 (2008–09).

For the dementia group, T0 could be wave 2 (2004–05), wave 3 (2006–07), 4 (2008–09) or 5 (2010–11).

**Table 2:** Social behaviours in the dementia and comparison groups.

Monthly activity (% yes)	Dementia group (n = 142)			Comparison group (n = 4029)		
	T0	T1	T2	T0	T1	T2
Face-to-face contact	78 (54.9%)	44 (31%)	33 (23.2%)	3494 (86.7%)	3596 (89.3%)	3517 (87.3%)
Telephone contact	78 (54.9%)	51 (35.9%)	36 (25.4%)	3621 (89.9%)	3733 (92.7%)	3644 (90.4%)

Note: T0 is the pre-diagnosis wave, T1 is the wave dementia is reported and T2 is the post-diagnosis wave.

### 3.2 Social engagement

**Fig 2:**
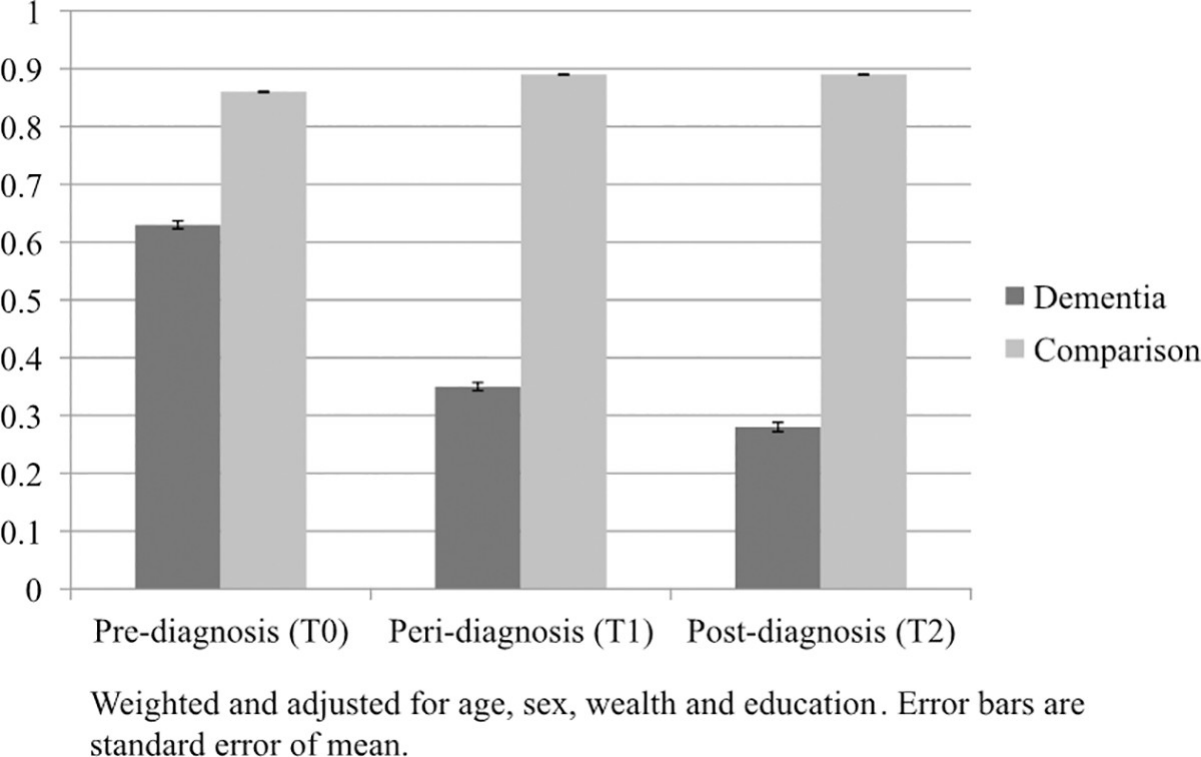
Proportion meeting friends & family on a monthly basis. Weighted and adjusted for age, sex, wealth and education. Error bars are standard error of mean.

*Face-to-face social contact*: This measure assessed whether there was a change in the proportion of participants meeting their friends or family on a monthly basis. We observed a main effect of group, whereby participants who developed dementia reported meeting friends and family less than the non-dementia group (*p <* 0.001). There was a significant group-by-time interaction effect for meeting friends and family (*p <* 0.001). As can be seen in Fig 2, there was a decrease in the proportion of those meeting friends and family in the dementia group from T0 to T1 and T2, with no corresponding reduction in the non-dementia group. We also looked within the dementia group alone to ascertain whether the difference in face-to-face social contact was significantly different between T1 and T2, as well as between T0 and T1. The fall in contact between T1 and T2 was significant (*p <* 0.001), as was the drop between T0 and T1 in the dementia group alone (*p <* 0.001).

**Fig 3:**
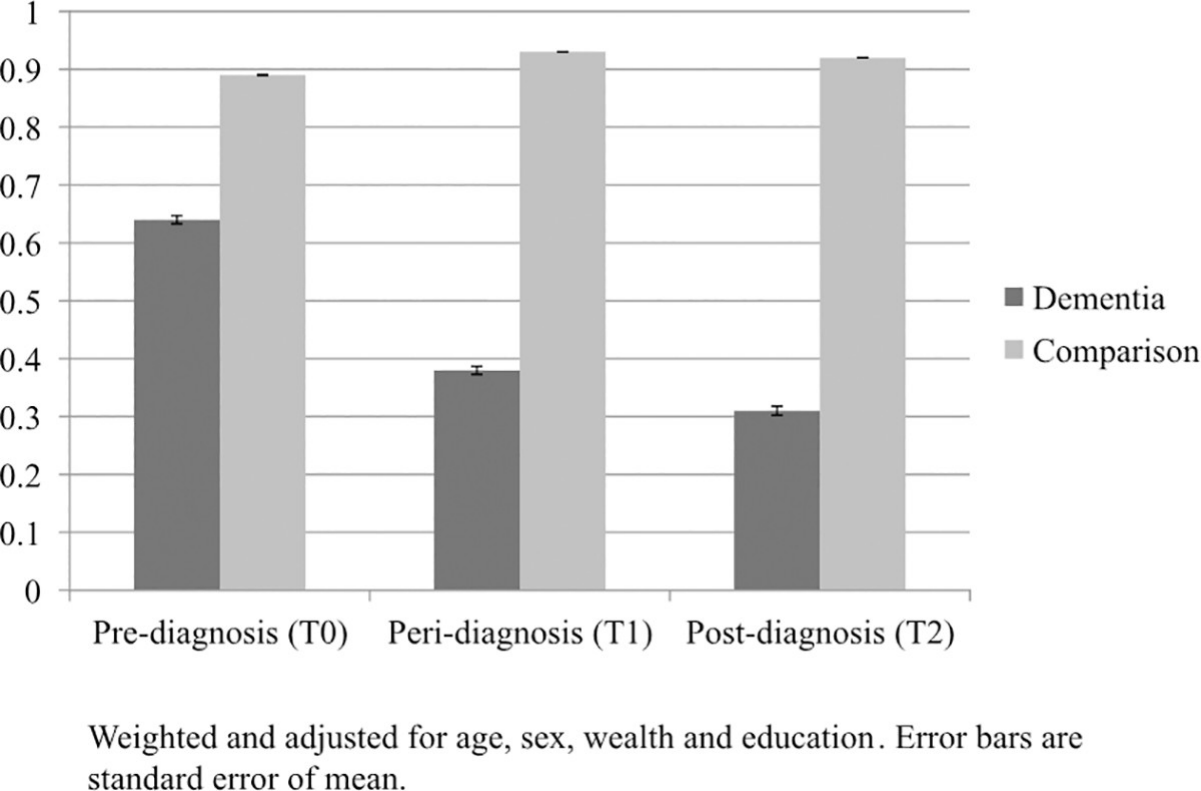
Proportion telephoning friends & family on a monthly basis. Weighted and adjusted for age, sex, wealth and education. Error bars are standard error of mean.

*Telephone contact*: We also assessed self-reported monthly telephone contact with friends or relatives (see Fig 3). There was a main effect of group for this measure, with lower levels of self-reported telephone contact in the dementia than the non-dementia group (*p <* 0.001). There was a significant group-by-time interaction (*p <* 0.001) for monthly telephone contact. There was a reduction in the proportion of participants in the dementia group reporting telephone contact with friends and family from the pre-diagnosis to the peri- and post-diagnosis stages. Contact in the non-dementia group remained fairly stable over the study period. Looking in the dementia group alone the fall in contact between T0 and T1 was significant (*p <* 0.001), as was the decline in contact between T1 and T2 (*p <* 0.001).

### 3.3 Sensitivity analyses

We conducted three sensitivity analyses to test the robustness of our findings. The first involved removing participants under 63 at T0 from the analyses, to account for the fact that dementia diagnoses (T1) before the age of 65 are uncommon and the fact that our dementia group were older on average at baseline than the non-dementia group (see Table 1) [16]. When we removed these younger participants from our dataset, there were 128 individuals in the dementia group and 2022 individuals in the non-dementia group, respectively. The mean age in the sample for this sensitivity analysis was 71.46 (0.13) years in the non-dementia group and 79.94 (0.52) years in the dementia group. The pattern of results remained unchanged removing younger participants from the analyses. We detected significant group-by-time interactions for both analyses (all *p* < 0.001).

To ascertain whether depressive symptoms or reports of another limiting illness could have influenced our findings, we add these variables as additional covariates in our models. Depression at baseline was a significant predictor of face-to-face (*p* < 0.001) and telephone contact (*p* < 0.001). However, the group-by-time interactions for both types of engagement remained significant following the inclusion of this covariate (*p* < 0.001). Limiting longstanding illness was not a significantly associated with face-to-face contact (*p* = 0.176), but was a significant predictor of telephone contact (*p* < 0.001) The inclusion of limiting longitudinal standing illness in the analysis did not alter the pattern of results.

We also tested whether the findings were consistent using a continuous measure for the frequency of social engagement. The findings for meeting friends and family are presented in S1 Fig. The pattern of results was consistent with the main analysis, with a significant group-by-time interaction (*p* < 0.001) detected with a reduction in face-to-face contact from the pre- to peri- diagnosis stage in the dementia group. We also looked at telephoning friends and family (see S2 Fig) and a significant group-by-time interaction was detected (*p* < 0.001), with a drop in contact following the diagnosis wave in the dementia group.

## 4. Discussion

This study shows for the first time the profile of social engagement in the years surrounding a diagnosis of dementia amongst adults aged 50 and above in a longitudinal population study. Adults who went on to develop dementia showed less social engagement 2 years prior to diagnosis. During the 4 years post-diagnosis, social engagement stayed unchanged, while those who developed dementia had further declines in social engagement.

Given that there were already baseline differences in social engagement two years prior to diagnosis, a central question is whether pre-clinical symptoms of cognitive decline were responsible for these differences, or whether people who were generally less socially engaged were more likely to develop dementia. Certainly, loneliness and social isolation appear to be independent predictors of dementia [24–27], and this could be related to the less frequent interaction with friends and family we captured in these analyses. However, it is notable that social engagement continued to decline across the 4 years post-diagnosis we tracked. Given that people who did not develop dementia had no change in their levels of social engagement with family and friend across this period, this suggests that certain behavioural and cognitive changes associated with dementia itself, also negatively affected social engagement.

In seeking to understand what these aspects are, at least three theories are plausible. The first is that direct effects of dementia associated impairments on memory and cognition mean that individuals forget how to engage socially, no longer remembering to attend meet up with friends or relatives or talk on the phone. For example, in relation to our findings about telephone contact with friends and family, studies of activities of daily living have previously shown that the ability to use a telephone starts to decline clearly around two years prior to the onset of dementia, which directly affects the ability of individuals to engage in this social behaviour [10]. Losing mental capabilities has also been found to threaten individuals’ sense of being a meaningful member of society [28]. The second theory is that changes in identity and a compromised sense of self as a result of dementia mean that individuals with dementia no longer gain as much pleasure from social activities [29]. Indeed, dementia is also associated with higher rates of depression, which itself is known to reduce social interaction [30]. The third theory is that changes in social engagement, in fact, occur in the immediate circle of friends and family of the individual. Negative social support is itself a risk factor for dementia as well as likely reducing the motivation for social contact with friends and family [31]. Studies have also shown that fear of dementia can reduce the interaction of older adults with friends who have developed dementia [32]. Changes in individual personal characteristics could mean that family and friends around them no longer wish to socialize with them. For example, selfishness is reported in half to two-thirds of AD and vascular dementia patients, while disinhibition is found in a third of AD and cerebrovascular dementia patients. Between 6 and 7 out of every 10 dementia patients also displays irritability and 97% of frontotemporal dementia patients display a loss of basic emotions, which could affect social interactions [8].

Whatever the reason behind the decline in social engagement found here, research suggests that it is important to encourage social engagement in people with dementia. A decline in social engagement is associated with a range of adverse neurobiological changes associated with faster cognitive deterioration, changes in memory, identity and mood that affect the quality of life of people with dementia and their family and carers. Therefore, there is an imperative need to consider how to increase social engagement. Personal communities created by formal and informal relationships can support a sense of belonging and identity for a person with dementia [33]. Social activities for people with dementia can support mental health [34]. Indeed, it is noted that a central strand within the UK Dementia Strategy is on the role of peer support in living well with dementia [35]. However, it is important that such support reaches those who could benefit the most. This research suggests that when an individual is diagnosed with dementia, there could be a value to exploring the range of social activities they are involved in and offering social prescriptions to encourage their engagement. Further, there could be a need to ensure adequate provision of dementia-friendly social activities in communities to facilitate supportive social engagement.

This study had a number of strengths. It used a large, nationally-representative sample of older adults in England and tracked two different types of social engagement. Through biennial data collection, we were able to track at every wave the time of dementia onset and compared individual's engagement for 2 years preceding and proceeding this diagnosis. We used inverse probability weighting using propensity scores to align dementia and non-dementia groups, adjusted for key demographic confounders, and conducted a number of sensitivity analyses to explore our assumptions; none of which materially affected our results. However, there are also some limitations. First, we analyzed dementia as a collective, but given previous research cited here showing different profiles of social engagement in people with different types of dementia (particularly frontotemporal dementia), it remains unknown whether social, behavioral changes are particularly evident in specific types of dementia. Further, we were reliant on self-reporting of dementia diagnosis and carer’s assessment of functional ability through validated measures, rather than more objective methods of diagnosis such as from a clinical diagnostic interview. Additionally, we assumed that all social engagement was beneficial. However, research suggests that social engagement can be positive or negative, with differential effects on health outcomes [31]. So the potentially different effects of positive and negative engagement remain to be explored further. The final sample differed significantly on key demographic characteristics from those present at wave 2 of ELSA who were not included in the study. Therefore, the sample may not be representative of the general population, and the findings may not apply to all groups. Finally, we only explored the two years either side of dementia diagnosis. As a result, the precise changes within these broad periods concerning social interactions and engagement remain known. And questions of the direction of causality between changes in social engagement and dementia onset remain to be explored further.

In conclusion, we presented for the first time a clear description in the level of social engagement prior to the onset of dementia and in the immediate years surrounding it. Given that social engagement is protective against the onset of dementia and supports the lived experience of people with dementia, it is important to find ways of promoting social interaction in older adults with and without dementia.

## Supporting information

S1 Fig Average rate of face-to-face contact (0 = no contact-4 = weekly contact). Weighted and adjusted for age, sex, wealth and education. Error bars are standard error of mean.
(TIF)

S2 Fig Average rate of telephone contact (0 = no contact-4 = weekly contact). Weighted and adjusted for age, sex, wealth and education. Error bars are standard error of mean.
(TIFF)

## Abbreviations

AD: Alzheimer’s disease
ELSA: English Longitudinal Study of Ageing
GEE: Generalized estimating equation
IQCODE: Informant Questionnaire on Cognitive Decline in the Elderly
