# Supplementary figures and images for: Social engagement before and after dementia diagnosis in the English Longitudinal Study of Ageing
Source: PLoS One. 2019 Aug 1;14(8):e0220195. doi: 10.1371/journal.pone.0220195 (PMC6675105; doi:10.1371/journal.pone.0220195)

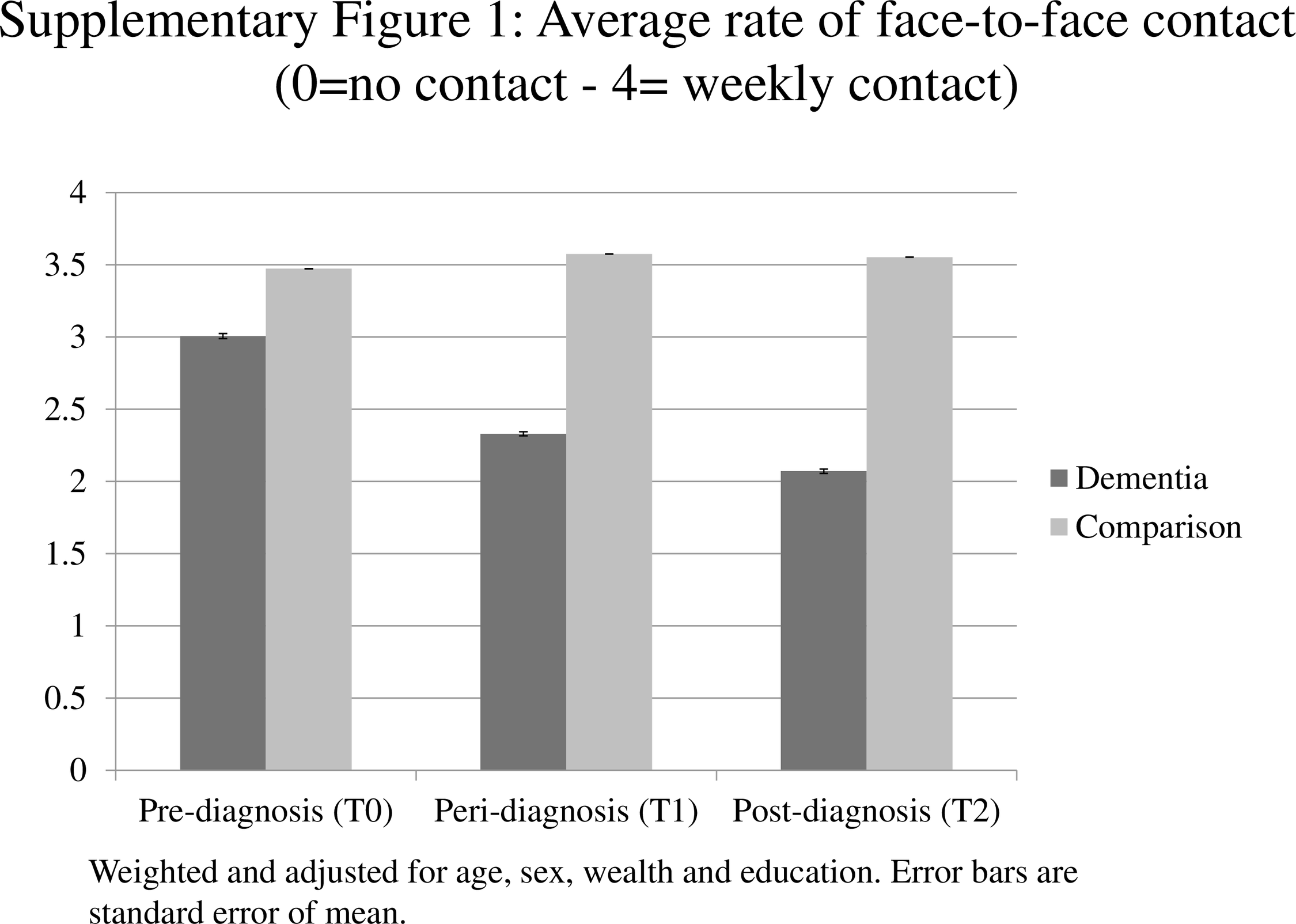

Supplement: S1 Fig — Weighted and adjusted for age, sex, wealth and education. Error bars are standard error of mean. (TIF) [file pone.0220195.s001.tif]

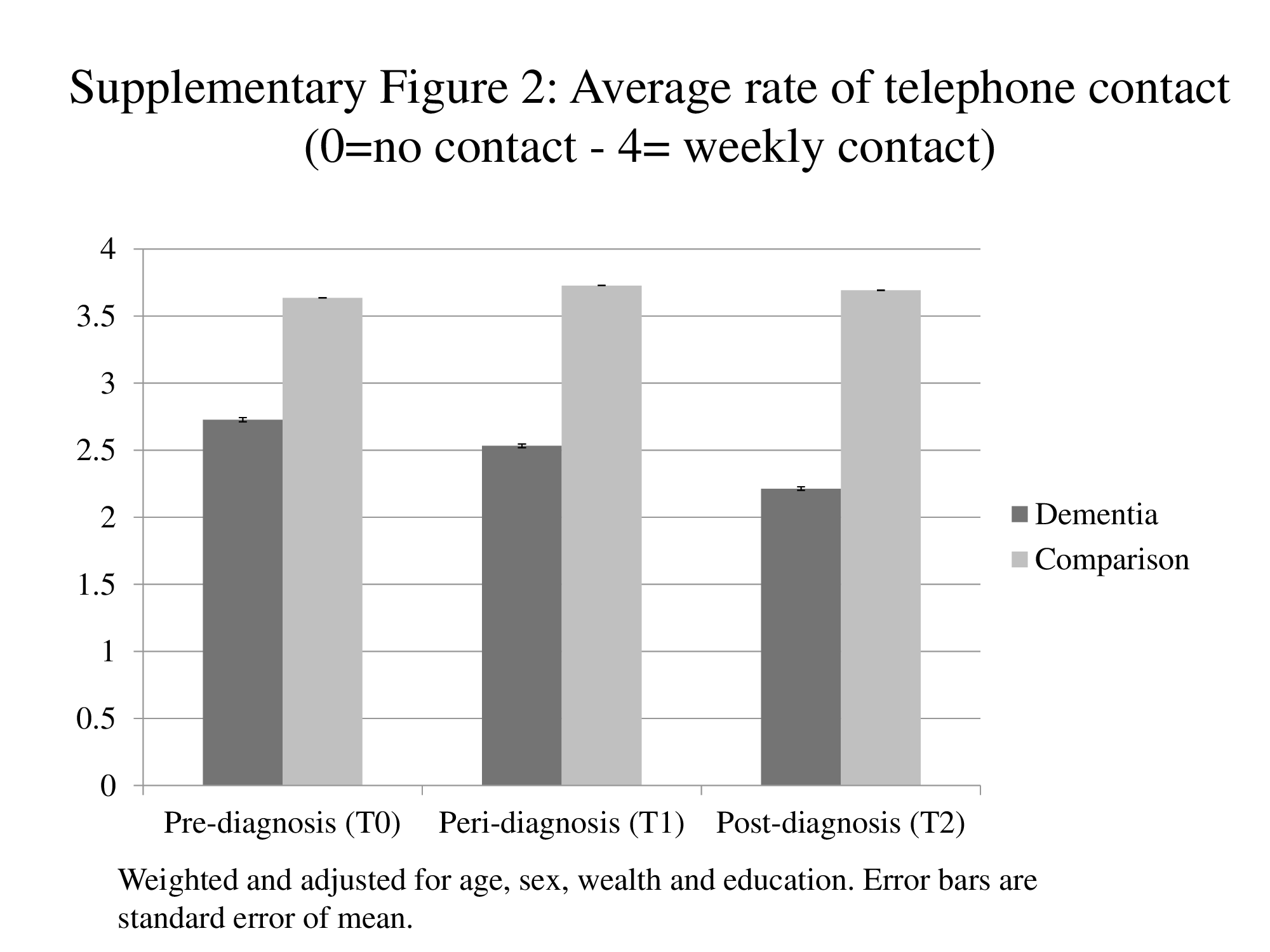

Supplement: S2 Fig — Weighted and adjusted for age, sex, wealth and education. Error bars are standard error of mean. (TIFF) [file pone.0220195.s002.tiff]
